# Sex-based and age-based differences in participation in an in-hospital atrial fibrillation screening study: a prospective cohort study in Switzerland

**DOI:** 10.1136/bmjopen-2025-112434

**Published:** 2026-03-30

**Authors:** Hildegard Tanner, Chrisoula Dernektsi, Felix Neugebauer, Eleni Goulouti, Anna Lam, Elena Elchinova, Nikolas Nozica, Helge Servatius, Fabian Noti, Andreas Haeberlin, Gregor Thalmann, Nikola Asenov Kozhuharov, Nicolas Rodondi, Drahomir Aujesky, Thomas Kueffer, Peter Jüni, Tobias Reichlin, Laurent Roten

**Affiliations:** 1 Department of Cardiology, Inselspital, Bern University Hospital, University of Bern, Bern, Switzerland; 2 Sitem Center for Translational Medicine and Biomedical Entrepreneurship, University of Bern, Bern, Switzerland; 3 Department General Internal Medicine, Inselspital, Bern University Hospital, University of Bern, Bern, Switzerland; 4 Institute of Primary Health Care (BIHAM), University of Bern, Bern, Switzerland; 5 Clinical Trial Service Unit and Epidemiological Studies Unit, Nuffield Department of Population Health, University of Oxford, Oxford, UK

**Keywords:** Pacing & electrophysiology, Mass Screening, Sexual and Gender Minorities, CARDIOLOGY

## Abstract

**Abstract:**

**Objectives:**

The under-representation of women in cardiovascular clinical trials is well documented but cannot be fully explained by sex-specific differences in disease prevalence. We investigated sex-related and age-related disparities in study participation within an atrial fibrillation (AF) screening study conducted among hospitalised patients.

**Design:**

Prospective cohort study.

**Setting:**

In-hospital patients in a single tertiary care centre in Bern, Switzerland.

**Participants:**

Patients aged 65–84 years evaluated for inclusion in the The SilenT AtRial FIBrillation cohort study, with inclusion stratified by sex and age.

**Interventions:**

Screening for AF using three consecutive 7-day Holter ECG recordings.

**Primary and secondary outcome measures:**

Prevalence of clinical and non-clinical exclusion criteria and participation rates among eligible patients, stratified by sex and age.

**Results:**

Of 11 470 patients evaluated, 10 675 were not enrolled. Clinical exclusion criteria were more prevalent among men than women (60.2% vs 50.5%, p<0.001), with prevalence increasing with age in both sexes. Consequently, fewer men met eligibility criteria compared with women (24.3% vs 32.6%, p<0.001). Among eligible patients, women were less likely to participate than men (20.2% vs 30.0%, p<0.001), and participation declined with advancing age in both sexes. Eligible men aged <75 years demonstrated the highest participation rate (34%).

**Conclusions:**

Significant sex-dependent and age-dependent disparities exist in both the prevalence of clinical exclusion criteria and participation rates among eligible patients in an AF screening study. These differences should be carefully considered in the design and planning of future clinical studies to improve representativeness.

**STRENGTHS AND LIMITATIONS OF THIS STUDY**

- Recruitment of study participants was prospectively stratified by predefined sex and age categories with fixed subgroup targets.
- A comprehensive screening log was maintained for all patients assessed for eligibility, allowing systematic analysis of exclusion and participation patterns.
- Clinical and non-clinical exclusion criteria were recorded according to a predefined hierarchical framework.
- Reasons for non-participation among eligible patients were not systematically collected beyond documented unwillingness to participate.
- The single-centre design and inclusion of in-hospital patients only may limit generalisability to other healthcare settings.

## Introduction

 Atrial fibrillation (AF) is the most frequent cardiac arrhythmia and is strongly age-related. Men have a higher age-adjusted risk of AF and tend to develop it earlier than women.^1^ However, the absolute number of men and women with AF is similar due to women’s longer life expectancy. AF is a major cause of ischaemic stroke, and oral anticoagulation is highly effective in preventing stroke in at-risk patients with AF. The guidelines of the European Society of Cardiology recommend screening for AF in patients aged ≥75 years, or those at high risk of stroke, with treatment of those who screen positive.^2 3^

**Figure 1:**
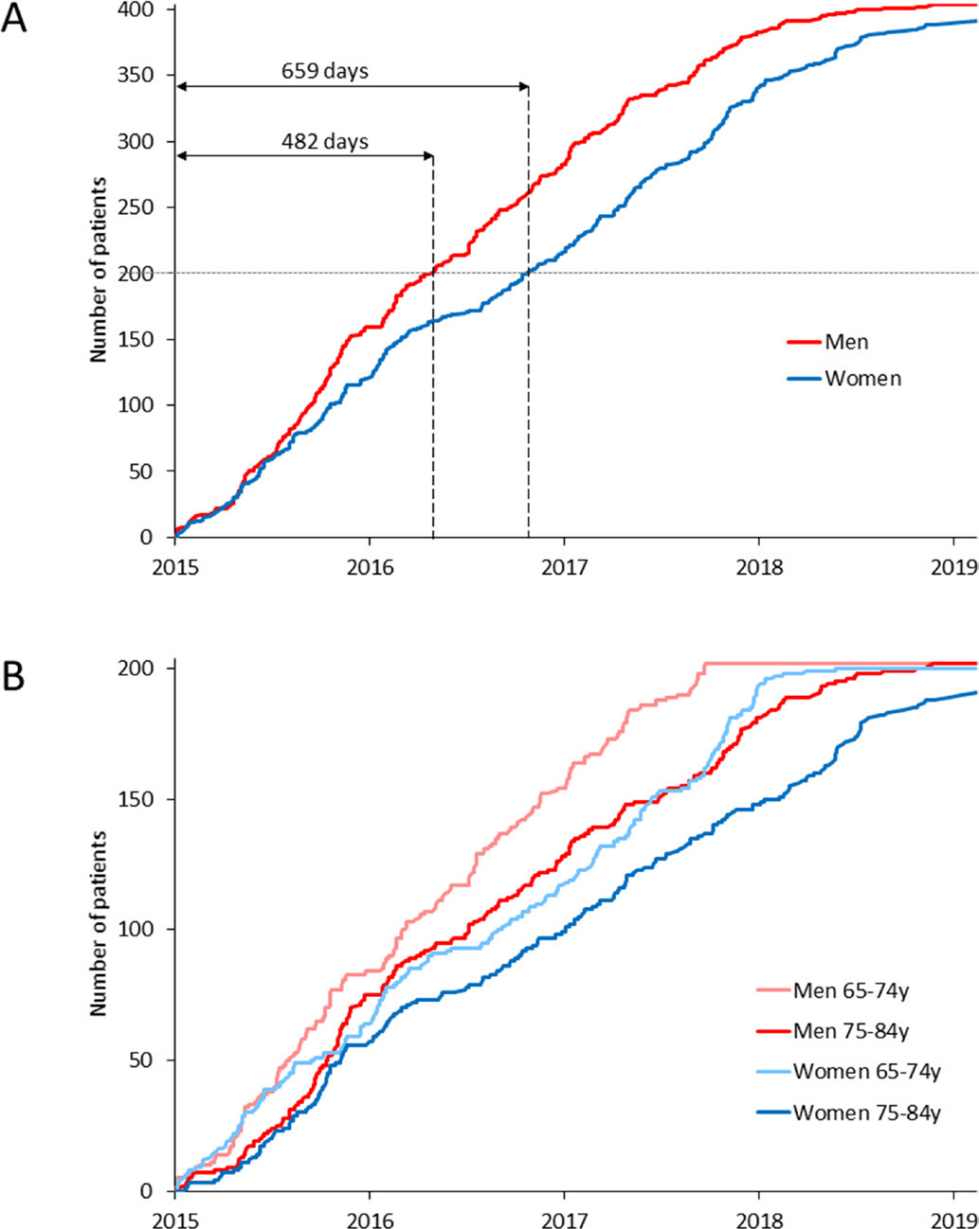
Cumulative study recruitment progress. (**A**) Illustrates the cumulative recruitment of male and female participants over time, while (**B**) further stratifies recruitment progress by sex and age groups (65–74 years and 75–84 years). The time required to reach 50% of the target population was 482 days for men and 659 days for women.

The SilenT AtRial FIBrillation (STAR-FIB) cohort study aimed to include equal numbers of women and men across four age groups between 65–84 years for screening of silent AF.^4^ Recruitment of women and older age groups proved more challenging than men and younger age groups (figure 1 and online supplemental figure). The reasons for this difficulty remain unclear, and data on potential sex-related and age-related differences in study exclusion reasons are limited.

The under-representation of women in cardiovascular clinical trials is well documented but cannot be fully explained by sex-specific differences in the prevalence of cardiovascular disease.^5^ Consequently, women often do not benefit from the same level of evidence in cardiology as men, leading to guideline recommendations that are frequently based on scientific data derived predominantly from men. Given established sex differences in cardiovascular structure and function, the inclusion of balanced numbers of women and men in cardiovascular trials is essential. To achieve this goal, sex-specific barriers that impede equal inclusion of sex categories should be recognised during the design phase of clinical trials, and appropriate counter-measures should be implemented.

The present study aimed to evaluate possible sex-related and age-related differences between patients successfully recruited and those excluded from the STAR-FIB cohort study.^6^

## Methods

### Study design and setting

The STAR-FIB cohort study was a prospective, stratified cohort study conducted at the Department of Cardiology, Inselspital, Bern University Hospital, University of Bern, Switzerland—a tertiary care centre serving a large regional population. The study formed part of the larger STAR-FIB study programme, which was designed to determine age-specific and sex-specific prevalence of silent AF and to develop prediction models for undiagnosed AF in an older hospitalised population. Recruitment occurred between January 2015 and June 2019. The details of the study programme, including a comprehensive description of the recruitment process, have been described elsewhere.^4^

### Eligibility and recruitment

Participants were adults aged 65–<85 years admitted to hospital for any acute medical reason. Daily review of hospital admission lists was used to identify potentially eligible patients. A stratified sampling technique with prespecified quotas was implemented to ensure balanced representation across four age groups (65–<70, 70–<75, 75–<80 and 80–<85 years) and sex categories (female/male, as recorded in the medical record). Recruitment was limited to 100 participants per sex–age subgroup, for a total planned cohort of 800 participants.

Because recruitment of men aged <80 years progressed more rapidly than other subgroups, random calendar weeks were selected after 10 months to restrict further screening of this subgroup while continuing continuous screening and recruitment for the remaining sex–age strata. This quota-based and calendar-based approach was prespecified and implemented to prevent over-representation of more rapidly enrolling strata. Daily review of admission lists was used to identify eligible patients. Screening logs were maintained to document eligibility and recruitment flow.

Clinical and non-clinical exclusion criteria:

The following clinical exclusion criteria were applied at initial assessment:

1. Previous diagnosis of AF.
2. Indication for long-term anticoagulation therapy.
3. History of acute coronary syndrome, cardiac intervention or acute heart failure within the past 3 months.
4. Planned cardiac intervention.
5. Presence of a cardiac implantable electronic device (pacemaker, implantable cardioverter/defibrillator or implantable cardiac monitor).
6. Use of class I or class III antiarrhythmic drugs.
7. Projected life expectancy of less than 1 year.
8. Inability to provide written informed consent.

Non-clinical reasons for exclusion were documented during in-person evaluation by the study team and included:

- Discharge prior to enrolment visit.
- Language or communication barriers.
- Participation in another study.
- Death prior to enrolment visit.
- Other reasons.
- Unwillingness to participate in the study.

A screening log was maintained for all 11 470 patients assessed for eligibility. For each patient, the log recorded sex, age category and the primary exclusion reason based on a prespecified hierarchy of clinical criteria. If no clinical criteria were met, non-clinical reasons were recorded as applicable. In patients with neither clinical nor non-clinical exclusion criteria, the study was explained and written informed consent was sought. Declination of participation was recorded but did not require justification.

### Screening protocol

Consenting participants without known AF underwent three consecutive 7-day Holter ECG recordings at prespecified intervals as part of the silent AF screening protocol. AF episodes lasting ≥30 s were considered diagnostic. Relevant clinical and demographic data were collected at baseline for subsequent analysis.

### Patient and public involvement

Patients and representatives of the public were not involved in the design, conduct, reporting or dissemination plans of this study. The research questions, outcome measures and study procedures were developed by the clinical investigators and study team without formal consultation with patient or public advisory groups.

###  Ethical approval

The study complies with the Declaration of Helsinki and was approved by the locally appointed ethics committee of the Canton of Bern (KEK-BE 257/14). All participants provided written informed consent prior to enrolment. The authors had full access to the data and take responsibility for the integrity of the data and the accuracy of the data analysis.

### Statistical analysis

Baseline characteristics and exclusion categories are presented as absolute numbers and percentages. All analyses were stratified by sex (female/male) and predefined age categories (65–69, 70–74, 75–79 and 80–84 years).

Associations between sex and the presence of clinical exclusion criteria were assessed using Pearson’s χ² test. Differences across age categories were analysed using χ² tests for trend (Cochran-Armitage test), performed separately within each sex.

Eligibility rates and participation rates among eligible patients were calculated as proportions within each sex–age subgroup. Comparisons between women and men were performed using χ² tests. Age-related trends in participation were assessed using χ² tests for trend.

All tests were two-sided, and a p value<0.05 was considered statistically significant. Given the exploratory nature of the study, no adjustment for multiple comparisons was performed.

Associations between exclusion criteria, sex categories and age groups were analysed using χ² tests. Numbers and percentages of patients meeting each criterion and of participation outcomes are presented by subgroup. Two-sided p values <0.05 were considered statistically significant.

## Results

During the recruitment phase, there were 44 636 hospital admissions. Of these, 33 166 patients excluded from screening due to age outside the target range, completion of recruitment for their sex–age subgroup, admission during non-recruitment weeks or readmissions. A total of 11 470 patients aged ≥65 years and <85 years were assessed for eligibility, of whom 391 women (49.2%) and 404 men (795 patients in total) were ultimately included in the study (figure 1 and online supplemental figure 1). The target of 100 women aged ≥80 years could not be achieved within the recruitment time frame; only 91 were enrolled.

**Figure 2:**
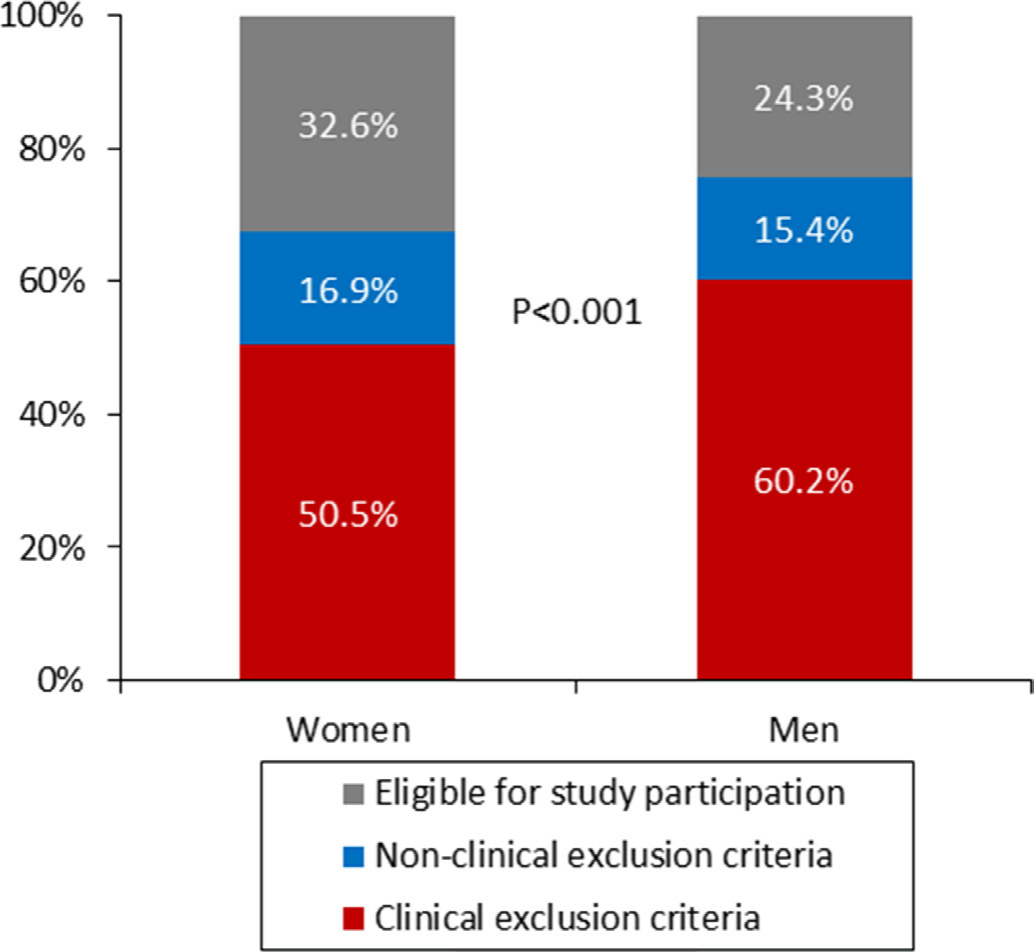
Distribution of patients by clinical exclusion criteria, non-clinical exclusion criteria and eligibility for study inclusion, stratified by sex.

**Figure 3:**
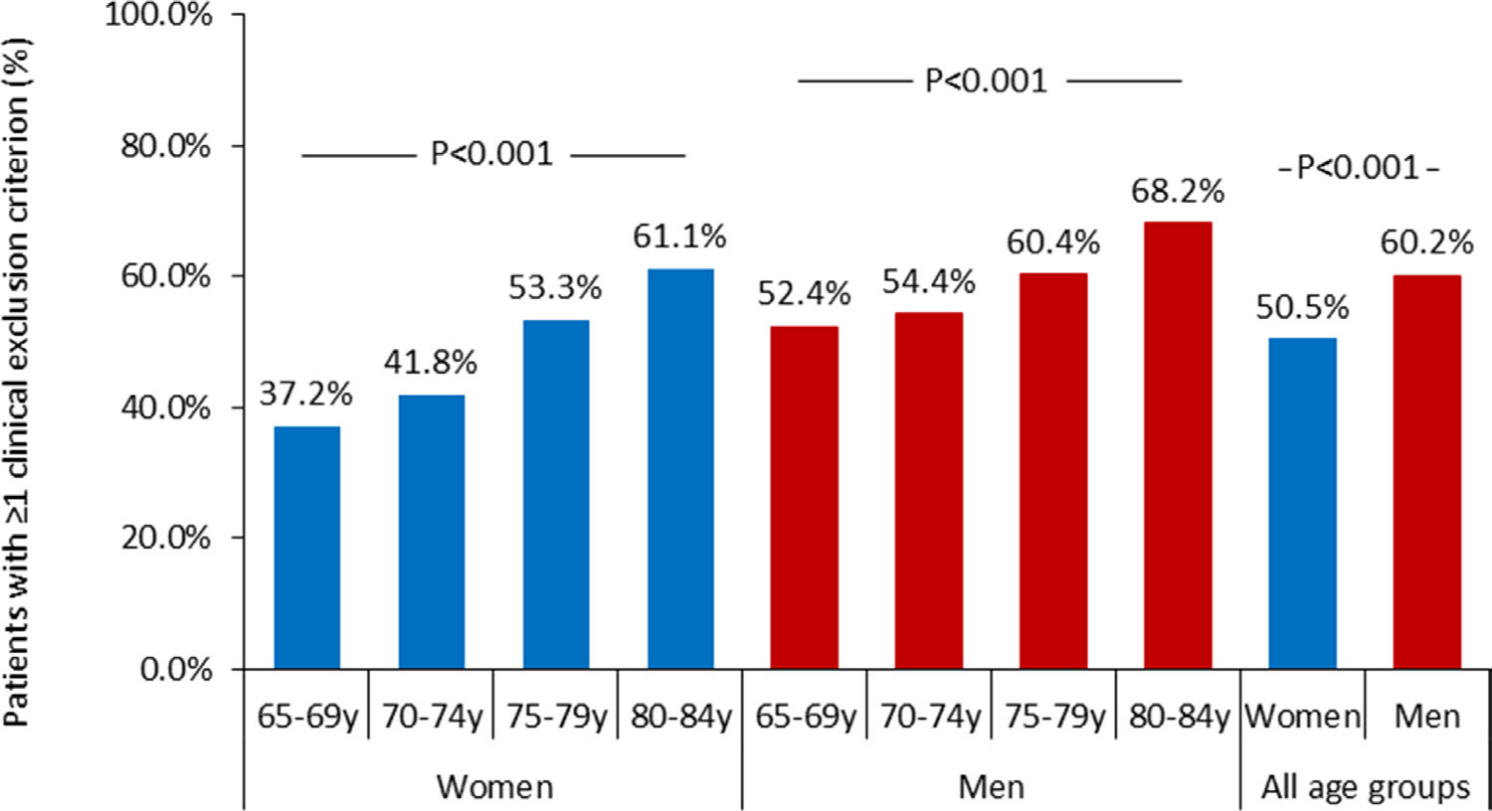
Bar chart showing the percentage of patients with at least one clinical exclusion criterion among all screened patients (denominator: all assessed patients within each sex–age subgroup). Age categories were 65–69, 70–74, 75–79 and 80–84 years. Percentages are displayed separately for women and men. The y-axis represents the proportion (%) of patients meeting ≥1 clinical exclusion criterion within each subgroup.

Of the 11 470 patients assessed for study eligibility, 10 675 patients were not enrolled. Table 1 provides an overview of the clinical and non-clinical reasons for exclusion, along with the total number of patients meeting the inclusion criteria, both overall and stratified by sex. The corresponding data for all age subgroups are presented in the online supplemental table. Clinical exclusion criteria were more common in men than in women (60.2% vs 50.5%, p<0.001; figures 2 and 3). In both sexes, the prevalence of exclusion criteria increased with age (p<0.001 for both; figure 3). The higher rate of clinical exclusion criteria in men was primarily driven by a greater prevalence of AF, cardiac implantable electronic devices and a history of acute coronary syndrome, cardiac intervention or acute heart failure within the past 3 months (table 1). As a result, a smaller proportion of men met the study inclusion criteria compared with women (24.3% vs 32.6%; p<0.001; figure 2).

**Table 1:** Number of patients by exclusion criteria, eligibility and inclusion in the study, stratified by sex

	All N=11 470	Women n=5942	Men n=5528
Clinical exclusion criteria	6332 (55.2)	3002 (50.5)	3330 (60.2)
Previous diagnosis of AF	2525 (22.0)	1138 (19.2)	1387 (25.1)
Indication for long-term anticoagulation therapy	738 (6.4)	409 (6.9)	329 (6.0)
History of acute coronary syndrome, cardiac intervention or acute heart failure within the past 3 months	983 (8.6)	459 (7.7)	524 (9.5)
Planned cardiac intervention	535 (4.7)	268 (4.5)	267 (4.8)
Presence of a cardiac, implantable, electronic device	588 (5.1)	196 (3.3)	392 (7.1)
Use of class I or class III antiarrhythmic drugs	112 (1.0)	49 (0.8)	63 (1.1)
Projected life expectancy of less than 1 year	156 (1.4)	72 (1.2)	84 (1.5)
Inability to provide written informed consent	695 (6.1)	411 (6.9)	284 (5.1)
Non-clinical reasons for exclusion	1855 (16.2)	1002 (16.9)	853 (15.4)
Discharge prior to inclusion visit	951 (8.3)	527 (8.9)	424 (7.7)
Language or communication barriers	275 (2.4)	154 (2.6)	121 (2.2)
Participation in another study	179 (1.6)	97 (1.6)	82 (1.5)
Death prior to inclusion visit	112 (1.0)	48 (0.8)	64 (1.2)
Other reason	338 (2.9)	176 (3.0)	162 (2.9)
Remaining patients eligible for study inclusion	3283 (28.6)	1938 (32.6)	1345 (24.3)
Unwillingness to participate in the study	2488 (21.7)	1547 (26.0)	941 (17.0)
Included in the study	795 (6.9)	391 (6.6)	404 (7.3)

Shown are numbers with percentages in parentheses.

AF, atrial fibrillation.

**Figure 4:**
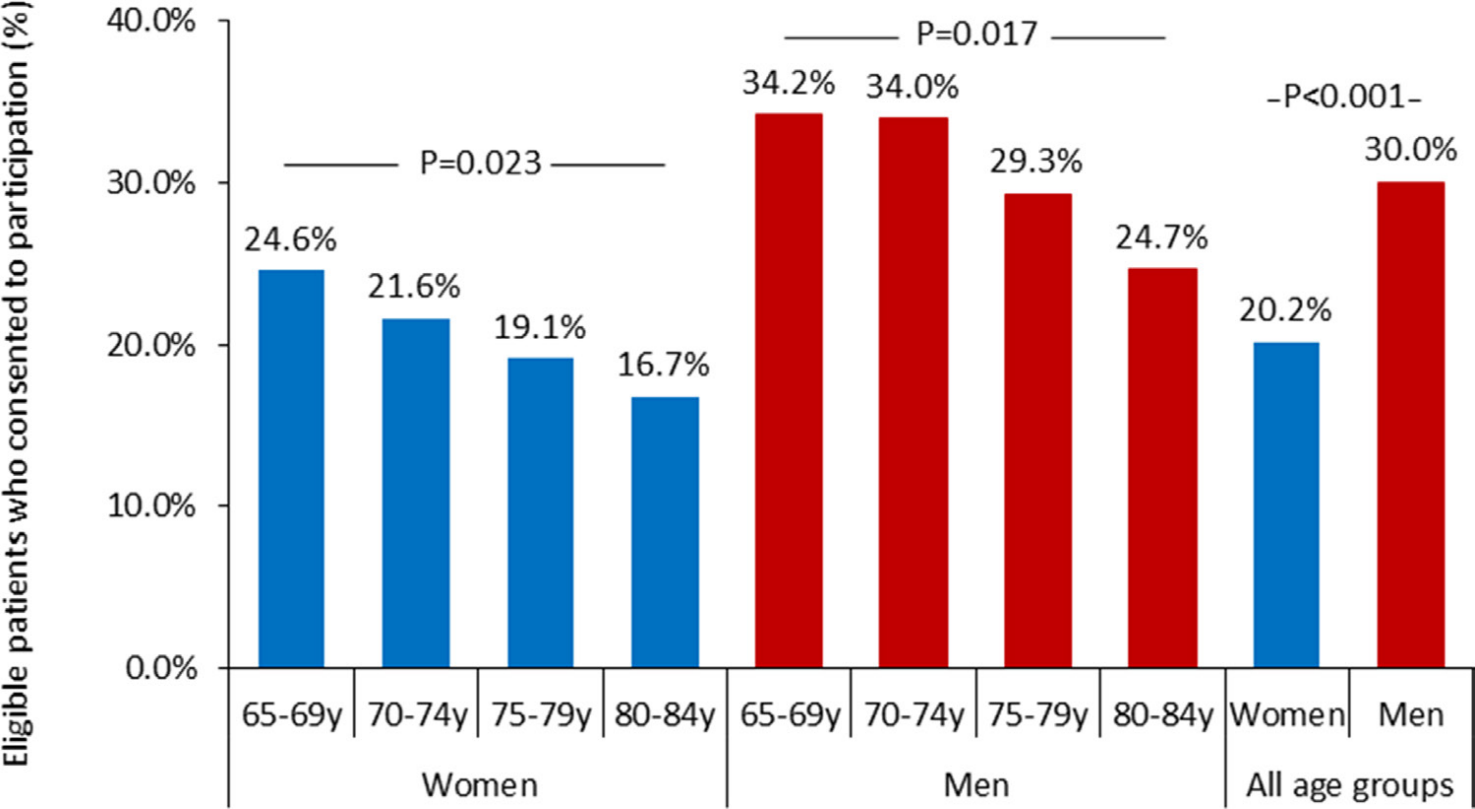
Bar chart showing—among eligible patients—the rate of patients who consented to be included in the study. The denominator includes only patients meeting all eligibility criteria (ie, those without clinical or non-clinical exclusion criteria). Age categories were 65–69, 70–74, 75–79 and 80–84 years. Results are stratified by sex. The y-axis represents the proportion (%) of eligible patients who consented to participate within each subgroup.

Among those meeting the eligibility criteria, women were less likely to participate than men (20.2% vs 30.0%; p<0.001; figure 4). In both sexes, participation rates declined with increasing age (p=0.023 for women and p=0.017 for men; figure 4). Eligible men under 75 years of age had the highest participation rate (34%). A graphical abstract summarising the primary sex-related and age-related differences in eligibility and participation for this study is presented in online supplemental figure 2.

## Discussion

The key findings of this study are:

- Clinical exclusion criteria were more common in men than in women and increased with age. Consequently, fewer men and older patients met the study inclusion criteria compared to women and younger patients.
- Among eligible patients, women were less likely to participate in the study than men.
- Participation rates declined with age in both sexes, with younger men showing the highest participation rates.

By design, this study aimed to recruit equal numbers of patients across four age groups for both sexes. While this design represents a strength of the study, it also underscores the challenges of achieving balanced recruitment across sex and age groups. Enrolling the first 200 women took 1.4 times longer than enrolling an equivalent number of men, despite efforts to address sex-based differences by curtailing the enrolment of younger men after the first 10 months. Even with an extended recruitment phase lasting over 4 years, it was not possible to achieve the prespecified target of 100 women aged 80–84 years, and recruitment was concluded after enrolling 91 participants in this subgroup.^4^ Without a sex-dependent and age-dependent inclusion strategy, women and patients over 80 years of age would have been significantly under-represented.

The importance of achieving adequate representation of women in clinical trials has been highlighted in a recent call-to-action published in the *European Heart Journal*.^7^ It is often assumed that the under-representation of women in clinical trials is due to the lower prevalence of cardiovascular disease compared with men. However, even after adjusting for sex-specific prevalence, studies in heart failure and coronary artery disease consistently report a disproportionally low percentage of female participants.^8 9^

Gong *et al* documented an increase in female enrolment in major cardiovascular randomised clinical trials over time, from 21% between 1986–1990 to 33% between 2011–2015.^10^ Despite this progress, the proportion of women enrolled varied significantly by disease type, with 37% in non-coronary artery vascular trials, 30% in coronary artery trials and only 28% in both heart failure and arrhythmia trials. These figures remain significantly lower than the expected proportion based on disease prevalence. Interestingly, the review found that trials reporting statistically significant results enrolled fewer women than those with non-significant findings. These findings emphasise the need for deliberate efforts to ensure equitable participation by sex, which can and should be achieved through intentional study design.

One crucial factor to consider during study planning is age-dependent and sex-dependent differences in the prevalence of exclusion criteria. For example, AF, as previously reported and demonstrated in our study, is more prevalent among men and increases with age.^11 12^ Additionally, men exhibit a higher prevalence of cardiac implantable electronic devices and recent cardiac events or interventions, resulting in a lower proportion of men meeting eligibility criteria for study participation. However, this difference was more than compensated for by the greater likelihood of eligible men consenting to study participation compared with women, particularly among those under 75 years of age. As a result, the inclusion rate of women was significantly lower, especially among those aged over 75 years.

The factors influencing the likelihood of eligible patients to consent to study participation remain poorly understood. Our study found lower participation rates among women and older patients, but patient-specific reasons for non-participation were not systematically recorded in the screening log. Several potential explanations may account for these patterns. For example, older participants and women, particularly those in older age groups, may have faced higher caregiving responsibilities, mobility limitations or concerns about the burden of repeated hospital visits and study procedures. Differences in attitudes towards research participation or prior experience with healthcare interventions may also play a role. As such, these observations are hypothesis-generating and should not be interpreted as definitive causal findings. Future studies that systematically collect patient-reported reasons for non-participation will be important to clarify these factors and guide strategies to improve equitable recruitment. In the context of our study, where patients were hospitalised for various medical reasons and invited to participate in an AF screening study, non-participation may have been influenced by general scepticism towards clinical research, concerns about the burden of study-related visits or examinations, or reluctance to commit additional time while managing their primary health condition. Specifically, the requirement for two additional hospital visits within 4 months after discharge and three repeated 7-day Holter ECG recordings likely posed a significant barrier in the STAR-FIB study, particularly for older or less mobile patients.

Trial-specific and patient-specific barriers to participation in cardiovascular trials have been previously studied, highlighting older age, female sex, longer trial duration and intensive trial-related testing as significant deterrents.^13^ While our study did not directly assess sex-specific and gender-specific factors, sociocultural dynamics may play a role. For example, women, especially in older age groups, may have ongoing caregiving responsibilities that reduce their availability for study participation, whereas men may have fewer such obligations postretirement. Additionally, factors such as educational level, income, social status and personal interest in science could further influence willingness to participate. Finally, the influence of sex concordance between patients and healthcare providers on clinical outcomes has been described in previous studies.^14 15^ However, such findings relate to clinical care settings and should not be directly extrapolated to research recruitment contexts. In our study, interactions occurred between potential participants and study personnel within a recruitment framework rather than a therapeutic relationship. Whether sex concordance between study personnel and potential participants influences willingness to participate remains unclear and warrants further investigation.

## Limitations

This study has several limitations. As a single-centre study conducted among hospitalised patients, its findings may not be generalisable to outpatient populations or other settings. A major limitation is the lack of detailed information on reasons for non-participation, restricting insights into patient-specific and trial-specific barriers to enrolment. Additionally, only binary sex categories (male/female) were recorded based on medical records, preventing an assessment of gender-based differences. No data on socioeconomic status and psychological factors were collected. The study focused on patients aged 65–84 years, limiting the applicability of findings to either younger or older populations. And finally, the findings of our study were not validated in other clinical trials. Future studies should address these limitations to provide a more comprehensive understanding of barriers to participation and ensure greater generalisability.

## Conclusions

The STAR-FIB study exemplifies the difficulties in recruiting older women and achieving balanced representation across sex and age groups in clinical research. Age-related differences were pronounced, with older patients facing higher exclusion rates due to clinical criteria and showing progressively lower participation rates, especially among women over 75 years. In contrast, younger men had the highest participation rates. Taken together, these findings underscore the need for recruitment strategies tailored to both age and sex to address these demographic differences and ensure representative generalisable clinical trials.

## Supplementary material

10.1136/bmjopen-2025-112434 online supplemental file 1

## Data Availability

The data that support the findings of this study are available from the corresponding author upon reasonable request.
