# Supplementary material for: Sex-based and age-based differences in participation in an in-hospital atrial fibrillation screening study: a prospective cohort study in Switzerland
Source: BMJ Open. 2026 Mar 30;16(3):e112434. doi: 10.1136/bmjopen-2025-112434 (PMC13052591; doi:10.1136/bmjopen-2025-112434)

Supplemental Material

Supplementary Table

Number of patients by exclusion criteria, eligibility, and inclusion in the study, stratified by sex and age category.

	Women 65-69y N=1057	Women 70-74y N=1300	Women 75-79y N=1603	Women 80-84y N=1982	Men 65-69y N=1057	Men 70-74y N=1184	Men 75-79y N=1403	Men 80-84y N=1884
**Clinical exclusion criteria**	**393 (37.2%)**	**544 (41.8%)**	**854 (53.3%)**	**1211 (61.1%)**	**554 (52.4%)**	**644 (54.4%)**	**847 (60.4%)**	**1285 (68.2%)**
Previous diagnosis of AF	111 (10.5%)	158 (12.2%)	350 (21.8%)	519 (26.2%)	172 (16.3%)	257 (21.7%)	358 (25.5%)	600 (31.8%)
Indication for long-term anticoagulation therapy	73 (6.9%)	94 (7.2%)	110 (6.9%)	132 (6.7%)	66 (6.2%)	72 (6.1%)	82 (5.8%)	109 (5.8%)
History of acute coronary syndrome, cardiac intervention, or acute heart failure within the past 3 months	82 (7.8%)	110 (8.5%)	130 (8.1%)	137 (6.9%)	128 (12.1%)	109 (9.2%)	129 (9.2%)	158 (8.4%)
Planned cardiac intervention	15 (1.4%)	44 (3.4%)	76 (4.7%)	133 (6.7%)	36 (3.4%)	47 (4.0%)	77 (5.5%)	107 (5.7%)
Presence of a cardiac, implantable, electronic device	26 (2.5%)	38 (2.9%)	50 (3.1%)	82 (4.1%)	71 (6.7%)	74 (6.3%)	92 (6.6%)	155 (8.2%)

Use of class I or class III antiarrhythmic drugs	12 (1.1%)	11 (0.8%)	16 (1.0%)	10 (0.5%)	16 (1.5%)	22 (1.9%)	12 (0.9%)	13 (0.7%)
Projected life expectancy of less than 1 year	18 (1.7%)	7 (0.5%)	17 (1.1%)	30 (1.5%)	18 (1.7%)	19 (1.6%)	25 (1.8%)	22 (1.2%)
Inability to provide written informed consent	56 (5.3%)	82 (6.3%)	105 (6.6%)	168 (8.5%)	47 (4.4%)	44 (3.7%)	72 (5.1%)	121 (6.4%)
**Organizational reasons for exclusion**	**257 (24.3%)**	**292 (22.5%)**	**226 (14.1%)**	**227 (11.5%)**	**211 (20.0%)**	**240 (20.3%)**	**208 (14.8%)**	**194 (10.3%)**
Discharge prior to inclusion visit	128 (12.1%)	144 (11.1%)	121 (7.5%)	134 (6.8%)	101 (9.6%)	122 (10.3%)	98 (7.0%)	103 (5.5%)
Language or communication barriers	46 (4.4%)	51 (3.9%)	31 (1.9%)	26 (1.3%)	40 (3.8%)	40 (3.4%)	28 (2.0%)	13 (0.7%)
Participation in another study	22 (2.1%)	30 (2.3%)	21 (1.3%)	24 (1.2%)	19 (1.8%)	22 (1.9%)	20 (1.4%)	21 (1.1%)
Death prior to inclusion visit	7 (0.7%)	11 (0.8%)	12 (0.7%)	18 (0.9%)	8 (0.8%)	10 (0.8%)	15 (1.1%)	31 (1.6%)
Other reason	54 (5.1%)	56 (4.3%)	41 (2.6%)	25 (1.3%)	43 (4.1%)	46 (3.9%)	47 (3.3%)	26 (1.4%)
**Remaining patients eligible for study inclusion**	**407 (38.5%)**	**464 (35.7%)**	**523 (32.6%)**	**544 (27.4%)**	**292 (27.6%)**	**300 (25.3%)**	**348 (24.8%)**	**405 (21.5%)**
Unwillingness to participate in the study	307 (29.0%)	364 (28.0%)	423 (26.4%)	453 (22.9%)	192 (18.2%)	198 (16.7%)	246 (17.5%)	305 (16.2%)
Included in the study	100 (9.5%)	100 (7.7%)	100 (6.2%)	91 (4.6%)	100 (9.5%)	102 (8.6%)	102 (7.3%)	100 (5.3%)

Shown are numbers with percentages in parentheses. AF, atrial fibrillation

Supplementary Figure 1

Cumulative study recruitment progress stratified by sex and age groups.

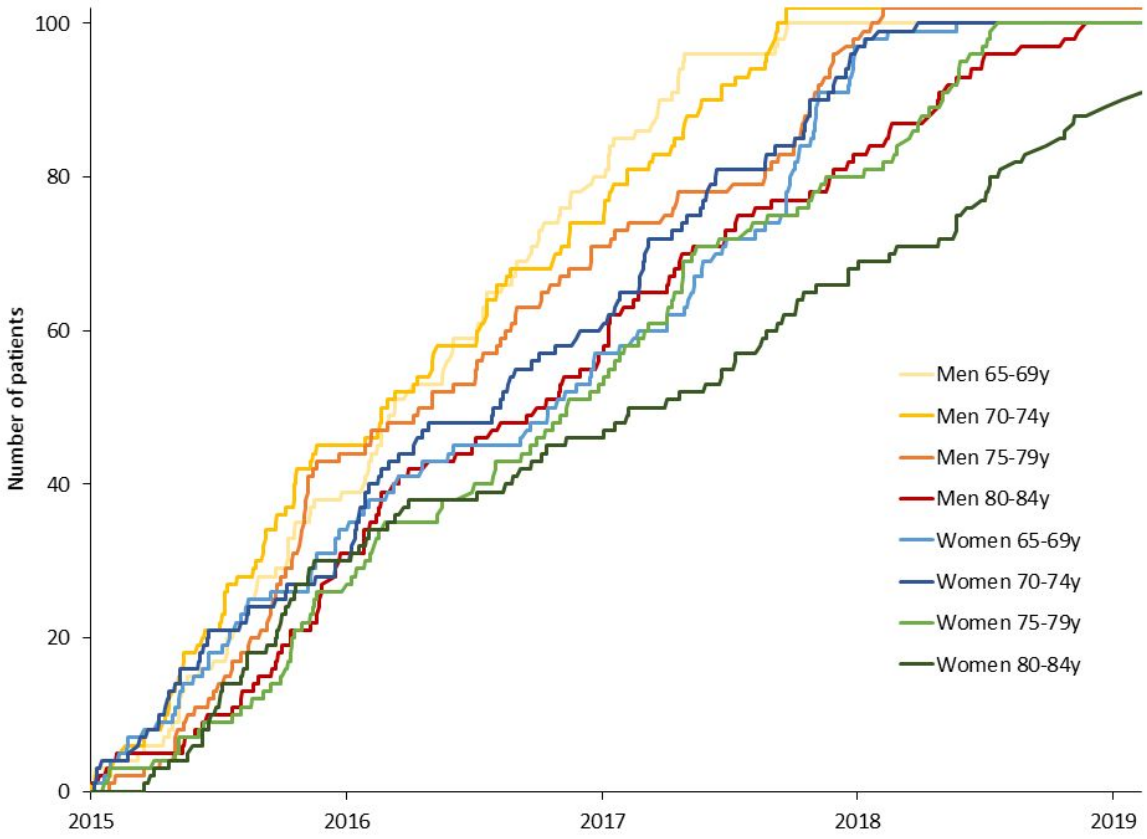

Supplementary Figure 2

The graphical abstract illustrates sex- and age-related differences in eligibility and participation rate in an in-hospital atrial fibrillation screening study.

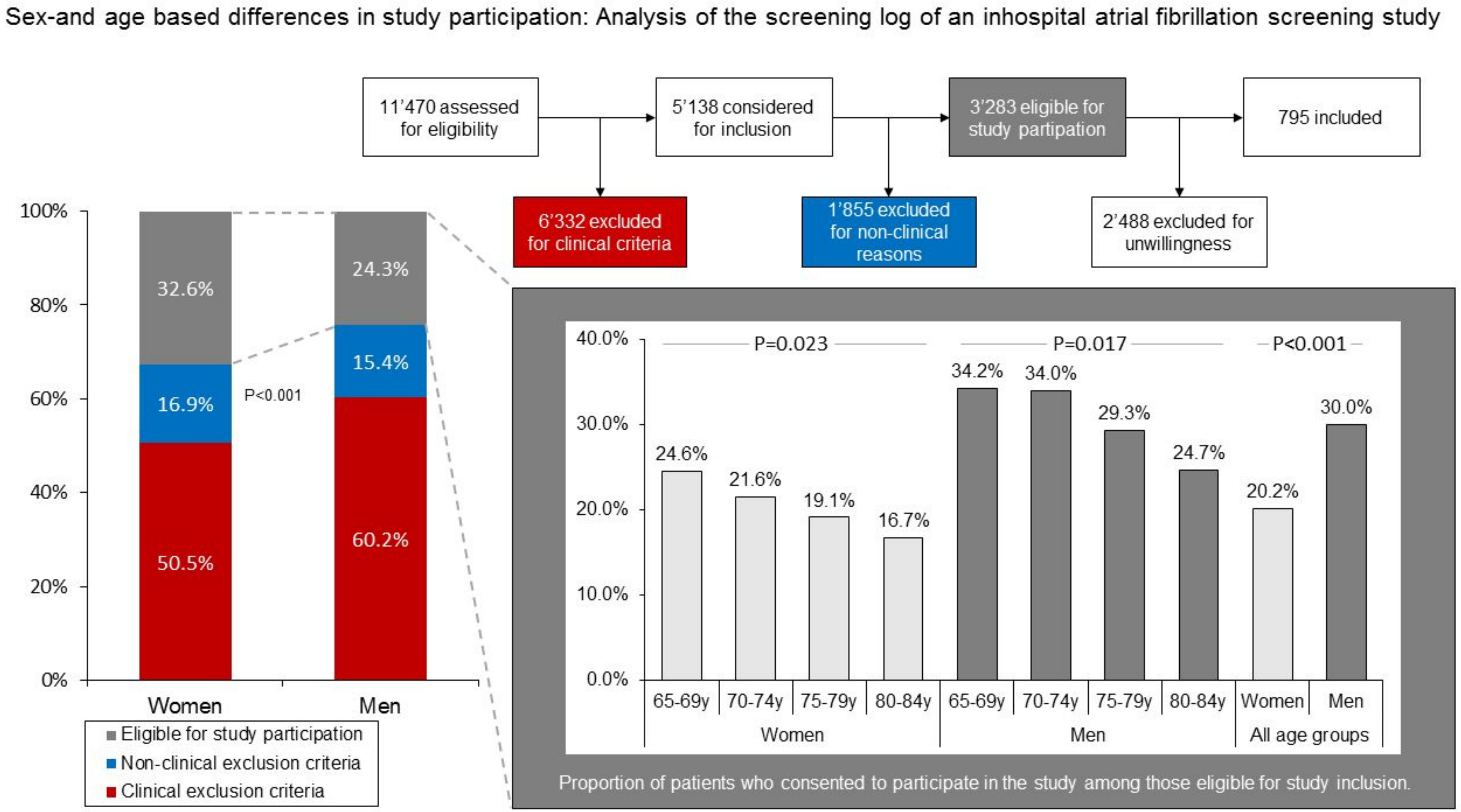

Supplement: online supplemental file 1 [file bmjopen-16-3-s001.pdf]
