# Supplementary material for: Sex-based and age-based differences in participation in an in-hospital atrial fibrillation screening study: a prospective cohort study in Switzerland
Source: BMJ Open. 2026 Mar 30;16(3):e112434. doi: 10.1136/bmjopen-2025-112434 (PMC13052591; doi:10.1136/bmjopen-2025-112434)
Supplement: Supplementary file 2 [file bmjopen-2025-112434.reviewer_comments.pdf]

PEER REVIEW HISTORY

BMJ Open publishes all reviews undertaken for accepted manuscripts. Reviewers are asked to complete a checklist review form and are provided with free text boxes to elaborate on their assessment. These free text comments are reproduced below.

ARTICLE DETAILS

Title (Provisional)

Sex- and age-based differences in participation in an in-hospital atrial fibrillation screening study: a prospective cohort study in Switzerland

Authors

Tanner, Hildegard; Dernektsi, Chrisoula; Neugebauer, Felix; Goulouti, Eleni; Lam, Anna; Nozica, Nikolas; Thalmann, Gregor; Kozhuharov, Nikola Asenov; Kueffer, Thomas; Jüni, Peter; Elchinova, Elena; Servatius, Helge; Noti, Fabian; Haeberlin, Andreas; Rodondi, Nicolas; Aujesky, Drahomir; Reichlin, Tobias; Roten, Laurent

---

VERSION 1 - REVIEW

**Reviewer**	**1**
**Name**	**Vinco, Giulia**
**Affiliation**	**University of Verona**
**Date**	**13-Nov-2025**
**COI**	**None**

-please check the reference format and make it consistent throught the manuscript (see line 292)

- I suggest to add clearer legends and more precise y-axis labeling on figures 3 and 4

- a flow-chart depicting patients evaluated and reasons for exclusion would be useful

- in the methods section you provide information that should be moved to the results section

- please clarify the statistical analysis section providing more information

- in the discussion, line 320-323: please distinguish between provider-patient outcome vs study personel-study recruitment setting

- among the limitations add: no data on socioeconomic status and psychological factors

---

**Reviewer**	**2**
**Name**	**Batra, Gorav**

**Affiliation**	**Uppsala University**
**Date**	**14-Feb-2026**
**COI**	**None**

In this study, the authors report sex- and age-related differences in eligibility and participation in an atrial fibrillation screening study. They found that, in this specific study, clinical exclusion criteria were more frequent in men. Among eligible patients, women were less likely to participate, however, the reasons for this were not captured. The study highlights the challenges of achieving balanced sex- and age-specific inclusion in clinical trials. The study is relevant and addresses an important evidence gap, but several issues should be addressed to further strengthen the manuscript.

1. The analysis relies on chi-square tests to assess associations between sex, age and participation. While this approach is appropriate for categorical data, the inclusion of multivariable analyses would strengthen the findings by allowing adjustment for potential confounders, such as comorbidities or reasons for hospitalisation. Without such adjustment, the observed differences may reflect underlying health differences rather than effects attributable to sex or age alone.

2. The findings may differ in other clinical trials. Therefore, an external validation based on data from another trial would strengthen the manuscript. If external validation is not possible, this limitation should be discussed in greater depth, and relevant findings from comparable studies should be discussed.

3. The manuscript reports lower participation among women and older patients but does not provide data on patient-specific reasons for non-participation. Although this is acknowledged, the discussion should in more detail raise potential explanations for these observations. Also, it should be clearly stated that these findings are hypothesis-generating rather than conclusive.

4. The study is single-centre and hospital-based. While this is explained in the discussion, further elaboration on how the findings may, or may not, be generalisable to other healthcare systems would be valuable.

5. The inclusion of a consort diagram showing eligibility, exclusions and participation stratified by sex and age would improve clarity and interpretation of the study population.

VERSION 1 - AUTHOR RESPONSE

Answers to reviewer 1:

Please check the reference format and make it consistent through the manuscript (see line 292)

Answer: The reference format was checked and the error of former line 292 corrected.

I suggest to add clearer legends and more precise y-axis labeling on figures 3 and 4

We agree, and added clearer legends to figure 3 and 4 including more precise y-axis labeling on both figures.

Figure 3

Prevalence of clinical exclusion criteria by sex and age group. Bar chart showing the percentage of patients with at least one clinical exclusion criterion among all screened patients (denominator: all assessed patients within each sex–age subgroup). Age categories were 65–69, 70–74, 75–79, and 80–84 years. Percentages are displayed separately for women and men. The y-axis represents the proportion (%) of patients meeting ≥1 clinical exclusion criterion within each subgroup.

Figure 4

Participation rate among eligible patients by sex and age group. Bar chart showing the percentage of eligible patients who provided written informed consent and were included in the study. The denominator includes only patients meeting all eligibility criteria (i.e., without clinical or non-clinical exclusion criteria). Age categories were 65–69, 70–74, 75–79, and 80–84 years. Results are stratified by sex. The y-axis represents the proportion (%) of eligible patients who consented to participate within each sex–age subgroup.

A flow-chart depicting patients evaluated and reasons for exclusion would be useful

Answer: We agree and added a supplementary figure 2 illustrating sex- and age-related differences in eligibility and participation including a flow-chart.

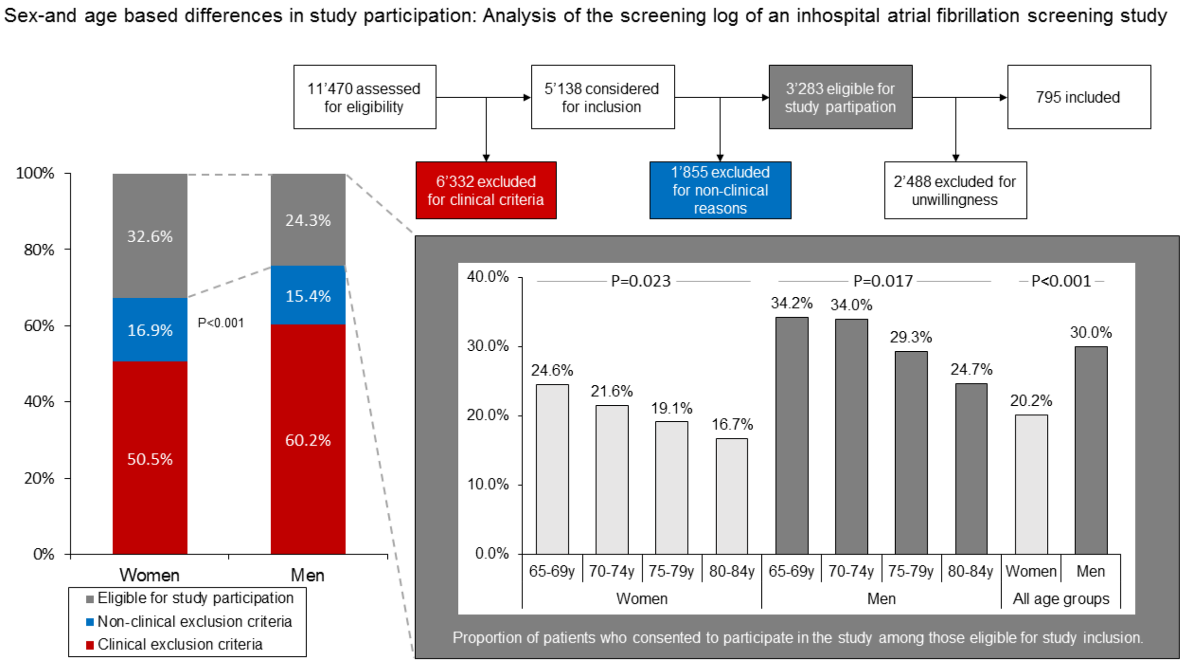

In the methods section you provide information that should be moved to the results section

Answer: We moved the following information from the methods section to the results section:

"During the recruitment phase, there were 44,636 hospital admissions. Of these, 33,166 patients excluded from screening due to age outside the target range, completion of recruitment for their sex-age subgroup, admission during non-recruitment weeks, or readmissions. A total of 11,470 patients aged ≥65 years and <85 years were assessed for eligibility, of whom 391 women (49.2%) and 404 men (795 patients in total) were ultimately included in the study (Figure 1 and Supplementary Figure 1). The target of 100women aged ≥80 years could not be achieved within the recruitment timeframe; only 91 were enrolled."

Please clarify the statistical analysis section providing more information

Answer: We clarified this section and provide the following additional information in the revised manuscript:

"Baseline characteristics and exclusion categories are presented as absolute numbers and percentages. All analyses were stratified by sex (female/male) and predefined age categories (65–69, 70–74, 75–79 and 80–84 years). Associations between sex and the presence of clinical exclusion criteria were assessed using Pearson's χ^2 test. Differences across age categories were analysed using χ^2 tests for trend (Cochran–Armitage test), performed separately within each sex. Eligibility rates and participation rates among eligible patients were calculated as proportions within each sex–age subgroup. Comparisons between women and men were performed using χ^2 tests. Age-related trends in participation were assessed using χ^2 tests for trend. All tests were two-sided, and a p value <0.05 was considered statistically significant. Given the exploratory nature of the study, no adjustment for multiple comparisons was performed."

In the discussion, line 320-323: please distinguish between provider-patient outcome vs study personel-study recruitment setting

Answer: Indeed with this very good comment from the reviewer and we corrected the discussion to:

"The influence of sex concordance between patients and healthcare providers on clinical outcomes has been described in previous studies. However, such findings relate to clinical care settings and should not be directly extrapolated to research recruitment contexts. In our study, interactions occurred between potential participants and study personnel within a recruitment framework rather than a therapeutic relationship. Whether sex concordance between study personnel and potential participants influences willingness to participate remains unclear and warrants further investigation."

Among the limitations add: no data on socioeconomic status and psychological factors

Answer: We agree and added the following sentence to the Limitations Section:

"Moreover, no data on socioeconomic status and psychological factors were collected."

Answers to reviewer 2:

In this study, the authors report sex- and age-related differences in eligibility and participation in an atrial fibrillation screening study. They found that, in this specific study, clinical exclusion criteria were more frequent in men. Among eligible patients, women were less likely to participate, however, the reasons for this were not captured. The study highlights the challenges of achieving balanced sex- and age-specific inclusion in clinical trials. The study is relevant and addresses an important evidence gap, but several issues should be addressed to further strengthen the manuscript.

Answer: We sincerely thank the reviewer for the thoughtful and balanced summary of our work. We appreciate the recognition that our study addresses an important evidence gap regarding sex- and age-related differences in eligibility and participation in clinical trials.

1. The analysis relies on chi-square tests to assess associations between sex, age and participation. While this approach is appropriate for categorical data, the inclusion of multivariable analyses would strengthen the findings by allowing adjustment for potential confounders, such as comorbidities or reasons for hospitalisation. Without such adjustment, the observed differences may reflect underlying health differences rather than effects attributable to sex or age alone.

Answer: We thank the reviewer for this important comment. We agree that multivariable analyses would strengthen the assessment of independent associations. However, the present study was based on the prospectively maintained screening log, which recorded only sex, age category, and the primary exclusion criterion using a predefined hierarchical approach. Detailed information on comorbidities or reasons for hospitalisation was not systematically collected for all screened patients, particularly those excluded early in the process. Because comprehensive clinical data were unavailable for the full screened population, multivariable adjustment for potential confounders was not feasible.

2. The findings may differ in other clinical trials. Therefore, an external validation based on data from another trial would strengthen the manuscript. If external validation is not possible, this limitation should be discussed in greater depth, and relevant findings from comparable studies should be discussed.

Answer: We thank the reviewer for this valuable suggestion. External validation was unfortunately not feasible, as our analysis was based on a prospectively maintained, study-specific screening log that captured recruitment and exclusion data in a structured and hierarchical manner. Comparable screening log datasets from other trials were not available to us.

We added the following sentence to the limitation section: "*And finally, the findings of our study were not validated in other clinical trials.*"

3. The manuscript reports lower participation among women and older patients but does not provide data on patient-specific reasons for non-participation. Although this is acknowledged, the discussion should in more detail raise potential explanations for

these observations. Also, it should be clearly stated that these findings are hypothesis-generating rather than conclusive.

Answer: We thank the reviewer for this important comment. As noted, patient-specific reasons for non-participation were not systematically collected in our screening log. We have now revised the Discussion to more fully explore potential explanations, such as higher caregiving responsibilities, mobility limitations, or perceived burden of repeated visits among women and older patients. We also clarified that, given the observational nature of our data, these findings are hypothesis-generating and should not be interpreted as conclusive.

We added the following sentences to the discussion:
"Our study found lower participation rates among women and older patients, but patient-specific reasons for non-participation were not systematically recorded in the screening log. Several potential explanations may account for these patterns. For example, older participants and women—particularly those in older age groups—may have faced higher caregiving responsibilities, mobility limitations, or concerns about the burden of repeated hospital visits and study procedures. Differences in attitudes toward research participation or prior experience with healthcare interventions may also play a role. As such, these observations are hypothesis-generating and should not be interpreted as definitive causal findings. Future studies that systematically collect patient-reported reasons for non-participation will be important to clarify these factors and guide strategies to improve equitable recruitment."

4. The study is single-centre and hospital-based. While this is explained in the discussion, further elaboration on how the findings may, or may not, be generalisable to other healthcare systems would be valuable.

Answer: We thank the reviewer for this comment. Our study was conducted in a single tertiary-care hospital with a prospectively maintained, study-specific screening log, which limits direct generalisability to other healthcare systems or outpatient settings. Therefore we prefer to not further elaborate on how the finding may or may not be generalisable to other healthcare systems. These limitations have already been addressed in the limitations section.

5. The inclusion of a consort diagram showing eligibility, exclusions and participation stratified by sex and age would improve clarity and interpretation of the study population.

Answer: We agree and added a supplementary figure 2 illustrating sex- and age-related differences in eligibility and participation including a flow-chart to improve clarity. As detailed sex and age specific information on exclusion critera is already given in the supplementary table, we prefer not to include an additional consort diagram.

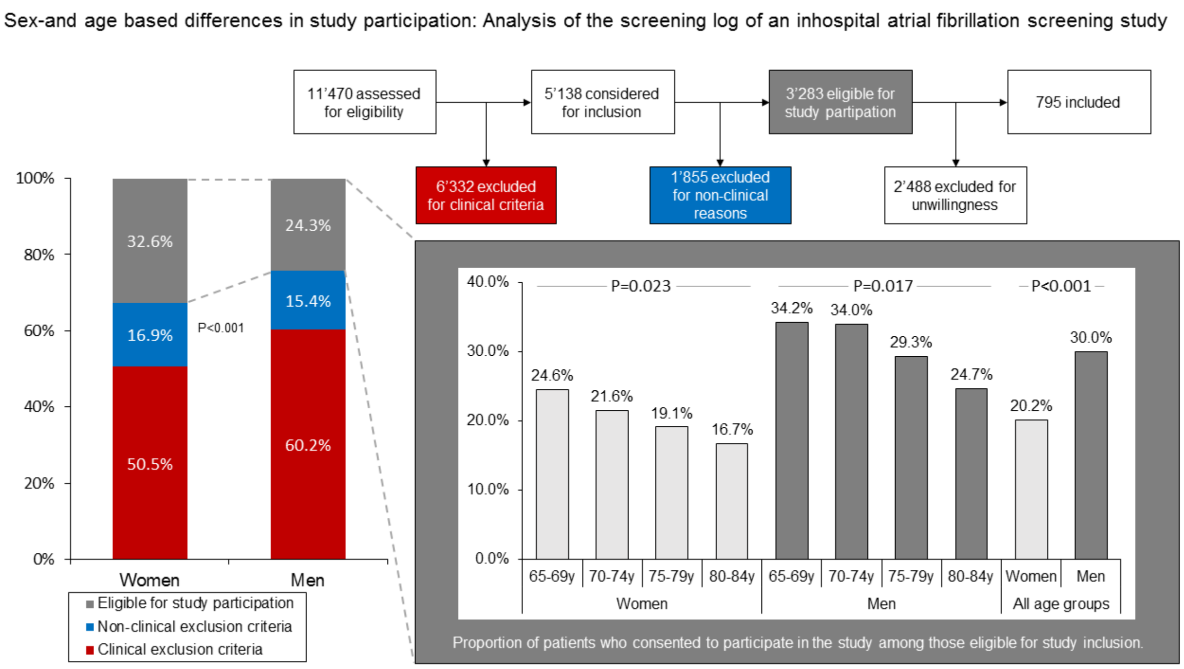

VERSION 2 - REVIEW

**Reviewer**	**2**
**Name**	**Batra, Gorav**
**Affiliation**	**Uppsala University**
**Date**	**12-Mar-2026**
**COI**	

The comments from the previous review round have been adequately addressed or responded to. I have no further comments. The conclusions are supported by the results, and the study's limitations are appropriately acknowledged.

VERSION 2 - AUTHOR RESPONSE

Answers to reviewer 1:

The comments from the previous review round have been adequately addressed or responded to. I have no further comments. The conclusions are supported by the results, and the study's limitations are appropriately acknowledged.

Answer: We thank the reviewer for the positive assessment. We appreciate the time and thoughtful review, which helped improve our manuscript.
